# A treat and extend protocol with Aflibercept for cystoid macular oedema secondary to central retinal vein occlusion – an 18-month prospective cohort study

**DOI:** 10.1186/s12886-020-01346-8

**Published:** 2020-02-24

**Authors:** Roderick O’Day, Noha Ali, Lyndell L. Lim, Sukhpal Sandhu, Thuy Chau, Sanjeewa Wickremasinghe

**Affiliations:** 1 Medical Retina Clinic, Royal Victorian Eye and Ear Hospital, Victoria, Australia; 2 Centre for Eye Research Australia, Royal Victorian Eye and Ear Hospital, Victoria, Australia; 3 Department of Ophthalmology, Assiut University, Assiut, Egypt

**Keywords:** Central retinal vein occlusion, Cystoid macular oedema, Anti-vascular endothelial growth factor inhibitors, Aflibercept, Treat-and-extend

## Abstract

**Background:**

To evaluate the safety and efficacy of a treat-and-extend protocol of aflibercept for cystoid macular oedema (CMO) secondary to central retinal vein occlusion (CRVO).

**Methods:**

Twenty patients with CMO secondary to CRVO were included in this prospective cohort study. After 3 loading 4-weekly injections, treatment intervals were increased by 2 weeks if there was no clinical activity, to a maximum of 12 weeks. If clinical activity recurred or persisted, the interval between injections was shortened by 2 weeks, to a minimum of 4 weeks. Main outcome measures were change in visual acuity and the proportion of patients gaining 15 or more Early Treatment of Diabetic Retinopathy Study (ETDRS) letters from baseline at 6, 12 and 18 months.

**Results:**

Mean BCVA gain from baseline was 19.7 ± 13.8, 22.2 ± 13.9 and 21.9 ± 15.8 ETDRS letters at 6, 12 and 18 months, respectively. Sixty-five percent of patients gained 15 or more ETDRS letters at 6 months, increasing to 70.6% at 12 and 18 months. Patients received 5.0 [4.0 to 6.0], 8.5 [8.0 to 10.3] and 11.0 [9.0 to 12.5] injections by 6, 12 and 18 months, respectively.

**Conclusions:**

The visual outcomes achieved with a treat-and-extend protocol in this study were similar to the pivotal trials of aflibercept for CMO secondary to CRVO, which used monthly and then as-needed protocols.

**Trial registration:**

Australian and New Zealand Clinical Trials Registry, registration number ACTRN12615000417583, 01/05/2015.

## Background

Cystoid macular oedema (CMO) is the most common cause of vision loss in patients with central retinal vein occlusion (CRVO) [1, 2]. Without treatment, CMO will resolve in only 30% of patients with non-ischaemic CRVO and less with ischaemic CRVO [2]. Intravitreal injections of anti-vascular endothelial growth factor (anti-VEGF) inhibitors have revolutionised the treatment of CMO secondary to CRVO, with mean gains of 3-lines (15 letters) in vision due to reduction in or resolution of CMO [3–8].

The optimal treatment regimen of intravitreal injections of anti-VEGF inhibitors is yet to be fully characterised. The pivotal studies – CRUISE [4, 5, 9] and COPERNICUS [3, 6, 10] – injected ranibizumab or aflibercept monthly for a loading period of six months and then as-needed when CMO recurred, with fixed follow up of monthly intervals during the first year and every three months in the second year. This resulted in excellent structural and functional improvements during the loading and monthly follow up periods, however, much of the visual gains were lost when the follow up interval was increased in the second year [3–6, 9, 10]. The GALILEO study evaluated aflibercept injections with a similar treatment regimen as COPERNICUS in the first year, but had 8-weekly follow up in the second year of the study, rather than 3-monthly. Nonetheless, the visual gains of the first year reduced in a similar fashion to COPERNICUS at the 18-month study end-point [7, 8, 11]. Since then, other trials have investigated different loading periods, follow up intervals and more recently, treat-and-extend protocols [12–16].

Treat-and-extend protocols have many potential benefits to both patient and clinician. They aim to achieve similar visual outcomes as more intensive regimens with less injections and clinics visits, as demonstrated in the management of neovascular age-related macular degeneration [17, 18]. It is well established that patients with prolonged duration of CMO secondary to CRVO have worse visual outcomes with intravitreal anti-VEGF therapy [3, 5]. Therefore, it is plausible that treating patients before CMO recurs in a treat-and-extend protocol may prevent irreversible structural damage to the retinal architecture that could limit long-term visual outcomes. In this prospective, single-center cohort study, we investigated the efficacy and safety of a treat-and-extend regimen with intravitreal aflibercept in patients with CMO secondary to CRVO over an 18-month period.

## Methods

This was a prospective, single-arm, cohort study conducted at the Royal Victorian Eye and Ear Hospital (RVEEH), Melbourne, Australia. The study was approved by the Human Research Ethics Committee of the RVEEH and adhered to the tenets of the Declaration of Helsinki. The study and its reporting adheres to the STROBE Statement for cohort studies.

Patients with treatment-naïve CMO secondary to CRVO and a baseline best corrected visual acuity (BCVA) of 20/40 to 20/400 (17 to 70 Early Treatment of Diabetic Retinopathy Study [ETDRS] letters) were included in the study. All patients who met the vision and central macular thickness (CMT) criteria without a relative afferent pupillary defect were included, irrespective of the amount of ischaemia found on fundus fluorescein angiography (FFA). Full inclusion and exclusion criteria are outlined in Table 1**.** Baseline retinal perfusion status was determined by FFA using the Central Vein Occlusion Study (CVOS) classification [1]. Patients were considered perfused if they had < 10 disc diameters of capillary non-perfusion.

**Table 1:** Inclusion and exclusion criteria

Inclusion Criteria	
Age ≥ 18 years	
CRVO with CMO as determined by fluorescein angiography and duration of onset less than 12 months	
BCVA of 17–70 ETDRS letters (20/40–20/400)	
CMT ≥300 μm as measured by OCT	
Absent relative afferent pupillary defect	
Exclusion Criteria	
Systemic	
Uncontrolled blood pressure (≥180 mmHg systolic and 110 mmHg diastolic)	
Chronic renal failure	
Major surgery within 1 month of study entry	
Previous systemic anti-VEGF treatment	
Women of childbearing age not using adequate contraception and women who are breast feeding	
Intercurrent severe disease such as septicaemia	
Ocular	
Glaucoma which is uncontrolled with IOP-lowering medications	
Past history of severe steroid response with IOP ≥ 35 mmHg following steroid treatment	
Loss of vision due to other causes (e.g. age-related macular degeneration, myopic macular degeneration)	
BCVA of < 20/200 in the fellow eye	
Argon laser photocoagulation within 3 months of study entry	
Previous intraocular surgery within 6 months of study entry	
Stroke or myocardial infarction less than 3 months prior to study entry	
Any active periocular or ocular infection or inflammation at screening or baseline.	
Subjects, who in the opinion of the Investigator, may not benefit from treatment due to pre-existing or current macular condition (eg, vitreomacular traction, epiretinal membrane, scar, foveal atrophy)	

*CRVO* central retinal vein occlusion, *CMO* cystoid macular oedema, *BCVA* best-crrected visual acuity, *ETDRS* Early Treatment of Diabetic Retinopathy Study, *CMT* central macular thickness, *OCT* optical coherence tomography, *IOP* intraocular pressure

Patients underwent BCVA assessment, intraocular pressure (IOP) measurement, slit-lamp examination and optical coherence tomography (OCT; Heidelberg Engineering, Heidelberg, Germany) at each visit. Gonioscopy was performed at baseline, 12 months and any visit after a decision was made to extend the treatment interval. In addition to these, color fundus photography and FFA were performed at the baseline and the month-12 visits. As part of the dilated fundus examination at each visit, venous closing pressure (CVP) was assessed by applying digital pressure on the eye while observing the retinal vessels at the optic disc. Patients were graded as having low CVP if their central retinal vein (CRV) collapsed before pulsation in the central retinal artery (CRA), medium if it collapsed at the same time as the CRA, and high if the CRV collapsed after the CRA or not at all.

### Treatment protocol

Treatment consisted of intravitreal injections of 2 mg of aflibercept (Eylea; Bayer Healthcare, Leverkusen, Germany) using a treat-and-extend protocol. An intravitreal injection of aflibercept was given at each study visit. All patients received three loading doses at 4-week intervals. After the loading period, the interval between study visits could be extended by 2 weeks at each visit, to a maximum of 12 weeks, if there was no clinical activity (see below for definition). If clinical activity was present at the end of the loading period, the interval between study visits was kept at 4 weeks until there was no clinical activity and then an attempt to extend the interval was made. Clinical activity was defined as any of:

1. CMO on OCT, classified as the presence of intraretinal fluid (IRF), subretinal fluid (SRF) or an increased CMT by 50 μm or more from the previous visit,
2. reduction in BCVA by 5 or more ETDRS letters from the previous visit (and at the third study visit only, an improvement in BCVA by 5 or more ETDRS letters from the second visit was considered clinical activity) and,
3. new retinal haemorrhages.

If clinical activity recurred during the attempt to extend the interval between study visits, it was reduced by 2 weeks. This was repeated until clinical activity was absent, or the interval between study visits was 4 weeks. The interval between study visits at which there was no further clinical activity, or 4 weeks, then became the maximum interval during the first year. A further attempt at extending the interval could be made during the second year on the first study visit at which there was no clinical activity. After the first year of the trial, if 12-weekly injection frequency was achieved treatment could be suspended, but clinical follow-up was continued*.* An attempt to extend the interval between study visits could also be made in the second year if clinical activity had persisted throughout the first year despite 4-weekly injections. This was continued as long as vision and CMT remained stable throughout the extension.

### Statistical analyses

The primary outcome measures were the change in BCVA from baseline and the proportion of patients gaining 15 or more ETDRS letters at 6, 12 and 18 months. Data was presented as the mean ± standard deviation (SD) when normally distributed or as median [interquartile range] (IQR) if not. Normality was assessed using the Shapiro-Wilks test. Descriptive statistics were performed of the baseline characteristics, visual and anatomical outcomes, treatment experience and venous closing pressure. The visual and anatomical outcomes were compared to the COPERNICUS and GALILEO trials in line graphs. To assess the predictive value of baseline characteristics on visual outcomes, a linear regression was applied. The dependent variable was change in ETDRS letters at 18 months from baseline. The independent variables were the measured baseline characteristics. For the regression analysis, baseline CMT was divided by 10. The resulting odds ratios were per 10 μm difference in baseline CMT. The difference in visual and anatomical outcomes at 18 months between patients that required a short or long interval between injections and less or more injections was assessed. For these analyses, patients were stratified into groups of less than the median, or, the median or more injection interval and number of injections. The homogeneity of variances between two groups was assessed using Levene’s test. Differences in continuous variables were compared using a student’s t-test or the Mann-Whitney U test for normally and not normally distributed data, respectively. Differences in proportions were analysed using Fisher’s exact test. All analyses were performed on a ‘per-protocol’ basis: patients lost to follow up were excluded from analyses at time points after they left the study.

A *P*-value of < 0.05 was considered statistically significant. All data were analysed using a commercially available software package (SPSS® 25; IBM Corporation, Armonk, NY, USA).

## Results

Twenty patients were enrolled in the study. Three patients were lost to follow up before the 12-month visit. Two of these withdrew because of coexistent medical conditions requiring hospitalisation and resultant difficulty in attending follow up appointments. One withdrew due to perceived lack of efficacy of treatment despite having improved from 60 ETDRS letters read at baseline (20/63) to 71 (20/40) at the last treatment visit (week 28). Consequently, all twenty (100%) patients completed the 6 month visit but only 17 (85%) patients completed the 12 and 18 month follow-ups, and their data were used for statistical analyses at those time points. One patient had laser photocoagulation and one had cataract surgery prior to study entry. The baseline characteristics of this cohort are presented in Table 2. The median baseline vision was 55.0 [36.8 to 64.8] ETDRS letters and the mean baseline CMT was 807 ± 238 μm. The mean age at study entry was 61.8 ± 14.3 years and 16 of 20 (80%) patients were male. The youngest patient was 23 years old, the next youngest was 46 years old. Due to the young age at presentation, the 23-year-old patient was referred to our physicians for review. Other than hypertension, no other systemic condition associated with CRVO was found. Investigations were performed for inflammatory, infectious and thrombophilic conditions. There was no difference in the measured baseline characteristics between those who completed follow up and those that did not. Similarly, there was no difference in the visual and anatomical outcomes between those who completed follow up and those that did not. (Supplementary Figure 1).

**Table 2:** Patient Demographics and Study Eye Characteristics of Patients with Central Retinal Vein Occlusion associated Cystoid Macular Oedema that were enrolled into the study

Characteristic	
Eye, n (%)	
Right	12 (60%)
Left	8 (40%)
Age, years	
Mean ± SD	61.8 ± 14.3
Range	23 to 84
Gender, n (%)	
Male	16 (80%)
Female	4 (20%)
Hypertension, n (%)	
Yes	10 (50%)
No	10 (50%)
Diabetes, n (%)	
Yes	7 (35%)
No	13 (65%)
Hypercholesterolemia, n (%)	
Yes	4 (20%)
No	16 (80%)
BCVA, ETDRS letter	
Median [interquartile range]	55.0 [36.8 to 64.8]
Range	20.0 to 70.0
> 20/200	15 (75%)
CMT, μm	
Mean ± SD	807 ± 238
Range	354 to 1281
IOP, mmHg	
Mean ± SD	14.3 ± 2.6
Perfusion status, n (%)	
Perfused	10 (50%)
Non-perfused	5 (25%)
Indeterminate	5 (25%)

*SD* standard deviation, *BCVA* best-correct visual acuity, *ETDRS* Early Treatment of Diabetic Retinopathy Study, *CMT* central macular thickness, *IOP* intraocular pressure

### Visual and anatomical outcomes

The mean BCVA was 73.7 ± 9.6 letters, 74.9 ± 8.9 letters and 74.7 ± 10.1 letters at 6, 12 and 18 months respectively. Thus, the mean gain in BCVA from baseline was 19.7 ± 13.8 ETDRS letters at 6 months, 22.2 ± 13.9 ETDRS letters at 12 months and 21.9 ± 15.8 ETDRS letters at 18 months.

Sixty-five percent of patients (13 of 20 patients) gained 15 or more ETDRS letters at 6 months, 70.6% (12 of 17 patients) at 12 months and 70.6% (12 of 17 patients) at 18 months. The mean change in the CMT was − 476 ± 312 μm at 6 months, − 542 ± 277 μm at 12 months and – 534 ± 300 μm at 18 months.

### Treatment experience

**Fig. 1:**
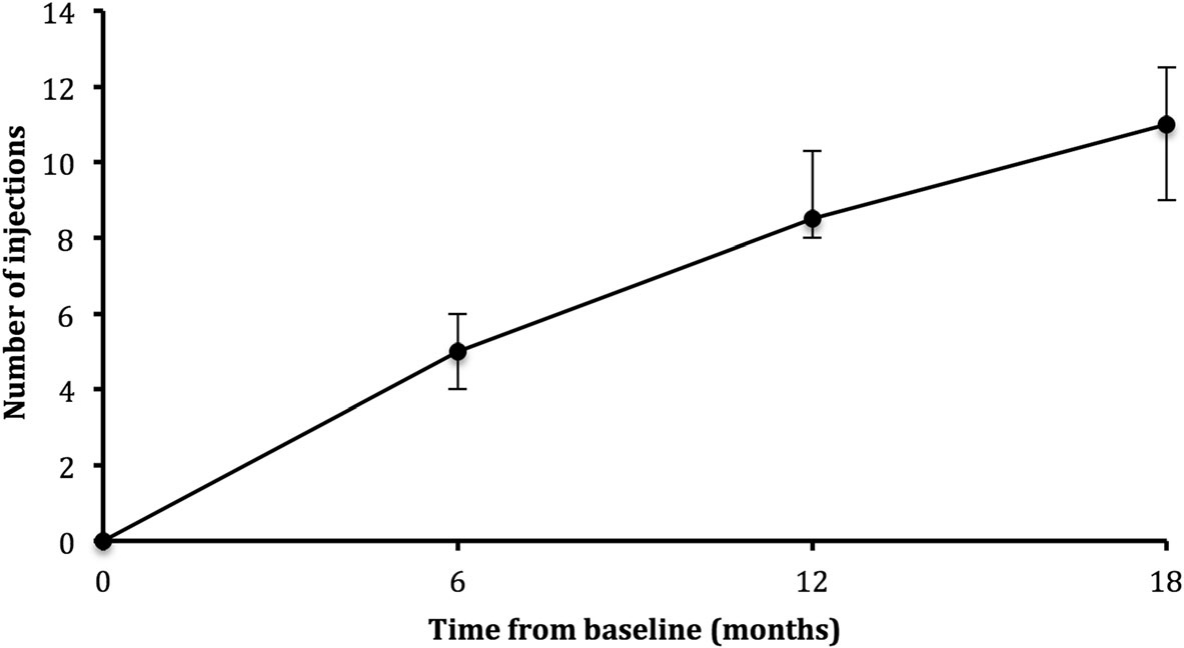
Median number of injections in our ‘treat and extend’ cohort at 6, 12, and 18 months. Vertical lines represent interquartile range

Patients received on average 5.0 [4.0 to 6.0] injections by 6 months (range 4 to 6), 8.5 [8.0 to 10.3] injections by 12 months (range 7 to 13) and 11.0 [9.0 to 12.5] injections by 18 months (range 8 to 16) in the study, Fig. 1. The minimum number of injections possible during the 18-month period of the trial was 7 and the maximum was 19. The median injection interval was 7.0 [6.0 to 8.0] weeks, 8.0 [6.0 to 12.0] weeks and 10.0 [6.0 to 12.0] weeks at 6, 12 and 18 months, respectively. The percentages of patients at each injection interval at 6, 12 and 18 months are shown in Table 3. During the first year, an attempt to extend the interval between injections was made in all patients. The median time from baseline at which patients first met criteria for the interval between injections to be extended was 12.0 [8.0 to 20.0] weeks (range 8 to 28). Seventeen of 20 (85%) patients were extended at or before the sixth study visit. In those that were extended, the median time at which first recurrence occurred was 30.0 [22.0 to 38.0] weeks (range 14 to 68), with the median interval being 8.0 [6.0 to 10.0] weeks (range 6 to 12).

**Table 3:** The Proportion of Patients at Each Injection Interval at 6, 12 and 18 months of Aflibercept Treatment

	6 months (*n* = 20)	12 months (*n* = 17)	18 months (*n* = 17)
Treatment Interval			
4 weeks	4 (20%)	4 (23.5%)	
6 weeks	6 (30%)	3 (17.6%)	5 (29.4%)
8 weeks	10(50%)	4 (23.5%)	3 (17.6%)
10 weeks		0 (0%)	4 (23.5%)
12 weeks		6 (35.3%)	5 (29.4%)

Three of 17 (17.6%) were successfully extended to a 12-week interval between injections without recurrence of disease activity by 12 months and thus it was possible to cease injections. These patients were followed and none experienced recurrent disease activity by 18 months. Of the patients who were unable to be extended without the development of recurrent CMO and reduction in BCVA during the first 12 months, 13 patients met the criteria for a second attempt to extend the interval between injections at the 12 month visit. In nine of these (69%), it was possible to extend the interval between injections, including 6 (46%) that did not have a further recurrence by 18 months.

### Comparison to COPERNICUS and GALILEO

**Fig. 2:**
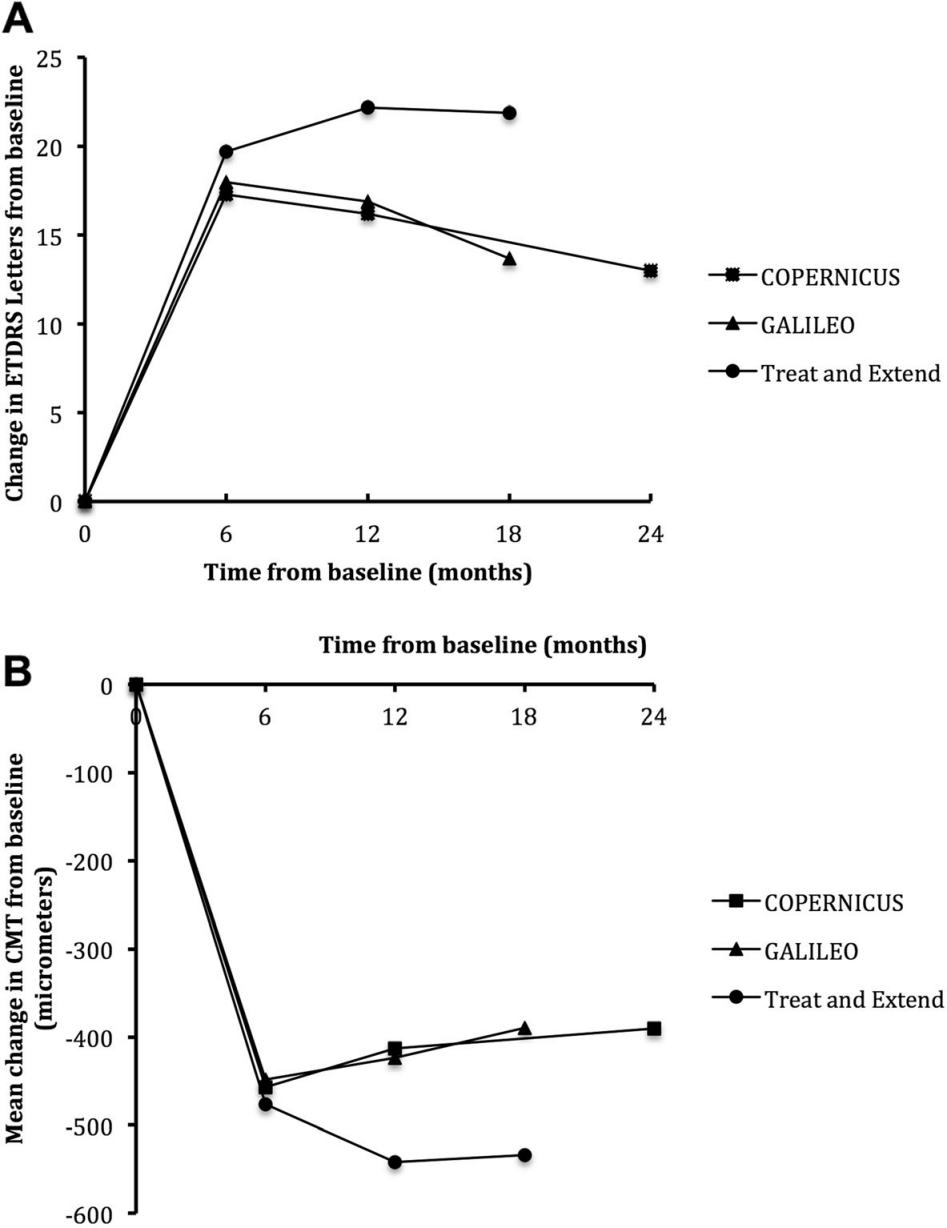
Comparison of the mean change in visual acuity (**a**) and mean change in central macular thickness (**b**) in our ‘treat and extend’ cohort from baseline with that of the COPERNICUS and GALILEO studies. ETDRS: Early Treatment of Diabetic Retinopathy Study; CMT: central macular thickness

In comparison with the COPERNICUS and GALILEO pivotal trials, the baseline characteristics of the patients in our study were well matched to the patients in those studies (Supplementary Table 1), although our patients had a higher baseline CMT and a smaller proportion of patients were considered to be perfused. Fig. 2 depicts the mean BCVA gain and the mean change in CMT in our patients as compared to COPERNICUS and GALILEO patients at month 6, 12 and 18 [3, 6–8, 10, 11]. The visual and anatomical results in this cohort appears to be similar to those of COPERNICUS and GALILEO during the monthly dosing period (6 months) of those studies and may be superior in the PRN periods (12 and 18 months). (Fig. 2) The number of injections patients received in our study was similar to those of COPERNICUS and GALILEO. In COPERNICUS, patients in the initial aflibercept arm received 6 loading doses and then 6.0 ± 3.4 injections from weeks 24 to 100 [7]. In GALILEO, patients in the initial aflibercept arm received 5.7 ± 0.9 injections between baseline and 24 weeks, 2.5 ± 1.7 injections between weeks 24 and 52 and 1.3 ± 1.1 injections between weeks 52 and 78 [10].

### Factors associated with outcomes

Eyes with poor baseline visual acuity (20/200 or less) had a greater proportion with a 15-letter gain and a similar rate achieving driving vision (20/40) at 18 months as compared to eyes with good baseline vision (20/80 or more). Four of 4 (100%) patients with baseline visual acuity of 20/200 or less had a 15-letter gain at 18 months versus 4 of 9 (44%) with a baseline visual acuity of 20/80 or more. Three of 4 (75%) patients with baseline visual acuity of 20/200 or less achieved driving vision (20/40) at 18 months versus 6 of 9 (66%) with a baseline visual acuity of 20/80 or more. Similarly, patients with worse baseline visual acuity had a greater improvement in vision at 18 months in the linear regression model. None of the other measured baseline characteristics, including perfusion status, was correlated to improvement in vision. (Supplementary Table 2).

The visual and anatomical outcomes after 18 months of treatment with aflibercept when stratified by the interval between injections is shown in Table 4.

**Table 4:** Visual and Anatomical Outcomes after 18 Months of Treatment with Aflibercept for Central Retinal Vein Occlusion, Stratified with Respect of Injection Interval at 18 Months

	Injection interval ″ 8 weeks	Injection interval ≥ 10 weeks	*P* value
Absolute visual acuity (ETDRS letters)	75.8 ± 11.5	73.8 ± 9.3	0.70
Mean gain in visual acuity (ETDRS letters)	22.8 ± 20.3	21.2 ± 11.6	0.85
Gain in visual acuity by ≥15 ETDRS letters	5 of 8 (63%)	7 of 9 (78%)	0.62
Absolute central macular thickness (μm)	262 [242 to 285]	272 [275 to 296]	0.54
Mean reduction in central macular thickness (μm)	− 552 ± 325	− 518 ± 296	0.83

*ETDRS* Early Treatment of Diabetic Retinopathy Study

Regardless of the injection interval or the number of injections required by 18 months visual and anatomical outcomes were similar in patients who required more intensive treatment (8 weeks) compared to those who needed less intensive treatment (≥10 weeks). Similarly, the number of injections given by 18 months also had no influence on visual or anatomical outcomes.

CVP was measured in 15 patients at the visit at which they first met the criteria to be extended; it was high in 9 (60%), medium in 1 (7%) and low in 5 (33%) patients. A reduction in CVP from the highest recorded prior to the time of first extension occurred in only 3 (20%) patients. At the visit at which clinical activity recurred, CVP was measured in 14 patients; it was high in 7 (50%), medium in 2 (17%) and low in 5 (33%). There had been an increase in CVP from the study visit prior to recurrence in 1 (7%) patient.

### Safety

There were no events of endophthalmitis, retinal tears or retinal detachments during the study period. Four serious adverse events (SAEs) occurred, none were related to study treatment. Two SAEs were reported in a 77 year-old female patient; a lumbar spine compression fracture 5 weeks after her 7th injection and an exacerbation of bronchiectasis 2 weeks after her 10th injection. A 72 year-old female developed microbial keratitis in the non-study eye 4 weeks after her 12th injection. A 57 year-old male patient had abdominal pain requiring admission 4 days after his 4th injection.

## Discussion

In this study, a ‘treat-and-extend’ regimen with aflibercept for CMO secondary to CRVO delivered similar visual and anatomical outcomes to those of the pivotal trials that investigated the same drug, COPERNICUS and GALILEO [3, 7]. Encouragingly, the loss of the visual and anatomical gains experienced in the second year of these studies did not occur in our patients [6, 8]. These results were achieved with a similar number of injections as the pivotal trials, but less clinic visits [3, 6–8]. At 18 months, the patients in this cohort had a mean improvement in visual acuity of + 21.9 (SD 15.8) ETDRS letters from baseline with a median of 11.0 [IQR 9.0 to 12.5] injections.

Treat-and-extend protocols have many potential benefits over monthly and pro re nata (PRN) regimens. Treating when the macula is dry may prevent long-term vision loss due to cumulative damage to photoreceptors and outer retinal structures that occurs when oedema is present, a pre-requisite for re-treatment in PRN protocols. The results of the pivotal trials are consistent with this: firstly, the sham-treated patients who were switched to anti-VEGF after 6 months in CRUISE, COPERNICUS and GALILEO did not achieve the same visual outcomes as those who were initially treated with anti-VEGF; and, secondly the gains achieved in these studies during the first 12 months were not maintained in the second year when the treatment protocols switched from monthly to PRN [2–10]. In addition to better visual outcomes, the number of clinic visits should be less in treat-and-extend protocols. In our study the median number of visits was 11.0 over 18 months, which would compare to 14 or 15 in the COPERNICUS study that had 3-monthly visits in the second year and 16 or 17 in the GALILEO study that had 8-weekly visits in the second year [2–7].

This study provides useful clinical information for patients and clinicians embarking on a treat-and-extend protocol for CMO secondary to CRVO, which, to date, has not been thoroughly investigated. Important findings were that almost one in four patients met the criteria to stop treatment at or before 18 months. Further, after 12 months of treatment, it is worthwhile attempting a first extension if clinical activity persists despite 4-weekly treatments or if clinical activity recurred during the first attempt to extend the interval between treatments. In almost 70% of these patients in our study, it was possible to increase the interval between injections, by an average of 2 weeks. Also, the amount of treatment that is required does not to appear to impact clinical outcomes. We found that there was no significant difference in visual or anatomical outcomes between those requiring less than the median, and the median or more, injections or interval between injections. This finding is in keeping with the theory that minimising the duration of oedema results in better visual outcomes for patients.

This study was novel in its standardised assessment of venous closing pressure at all study visits. Absolute and change in CVP was not linked to change in clinical activity; its absence and, therefore, the extension in the interval between injections, or its recurrence. This is in contrast to a previous report that found a prognostic significance of CVP in patients with CRVO [19], but is in keeping with our clinical experience of this being a poor marker for treatment outcomes and suggests that raised hydrostatic pressure is not the only driver for recurrent in CMO.

In comparison to other treat and extend studies in treating CMO as a result of CRVO, the Study of Comparative Treatments for Retinal Vein Occlusion 2 study randomised patients who responded well to 6, 4-weekly injections of aflibercept to a PRN or treat-and-extend protocol [12]. Our study differs from this in that all patients received a treat-and-extend protocol and we used a loading dose regimen consisting of three, 4-weekly injections. Casselholm de Salles et al. also recently published a prospective study comparing ranibizumab to aflibercept using a treat-and-extend protocol [13]. It varies from ours in that recurrence was defined as CMO on OCT and no further attempt to extend the interval between injections was permitted after a recurrence of fluid. The aflibercept arm in their study had very similar visual outcomes (mean improvement in visual acuity of 22.4 (15.3–29.4) at 18 months to ours and required the same number of injections (10.9). The similarity in outcomes adds to the validity of the results of both studies, performed in different countries with different patient populations. This is particularly pertinent given the relatively small numbers in each study.

The weaknesses of this study are its small numbers and the lack of internal validation as a result of its single-arm design. Accordingly, caution should be used when comparing our results to those of the pivotal clinical trials and in interpreting our safety data. Its strengths are its prospective design and single-center nature.

## Conclusions

In conclusion, our study suggests that a treat-and-extend protocol leads to similar visual results compared to the pivotal, COPERNICUS and GALILEO trials and avoided the visual decline that occurred during the second year of these trials. This was achieved with a similar number of injections, but less clinic visits. Thus treat-and-extend protocols for CMO secondary to CRVO, which are less taxing on patients and healthcare services, appear to be at least equivalent to monthly and then PRN regimens.

## Supplementary information

**Additional file 1.** Supplementary Table 1. Comparison of baseline characteristics between the current study and the pivotal trials: COPERNICUS and GALILEO.

**Additional file 2.** Supplementary Table 2. The Significance of Baseline Demographics and Study Eye Characteristics on Mean Change in Visual Acuity after 18 Months of Aflibercept Treatment.

**Additional file 3.** Supplementary Figure 1. Comparison of visual and anatomical outcomes in patients who completed follow up (A,C) and those that did not (B,D).

## Data Availability

The datasets used and/or analysed during the current study are available from the corresponding author on reasonable request.

## Abbreviations

Anti-VEGF: Anti vascular endothelial growth factor
BCVA: Best corrected visual acuity
CMO: Cystoid macular oedema
CMT: Central macular thickness
CRA: Central retinal artery
CRV: Central retinal vein
CRVO: Central retinal vein occlusion
CVOS: Central Vein Occlusion Study
CVP: Venous closing pressure
ETDRS: Early Treatment of Diabetic Retinopathy Study
FFA: Fundus fluorescein angiography
IQR: Interquartile range
OCT: Optical coherence tomography
PRN: Pro re nata
RVEEH: Royal Victorian Eye and Ear Hospital
SAE: Serious adverse event
SD: Standard deviation
