# Supplementary material for: A treat and extend protocol with Aflibercept for cystoid macular oedema secondary to central retinal vein occlusion – an 18-month prospective cohort study
Source: BMC Ophthalmol. 2020 Feb 24;20:69. doi: 10.1186/s12886-020-01346-8 (PMC7038604; doi:10.1186/s12886-020-01346-8)
Supplement: Supplementary file 1 — Additional file 1. Supplementary Table 1. Comparison of baseline characteristics between the current study and the pivotal trials: COPERNICUS and GALILEO. [file 12886_2020_1346_MOESM1_ESM.docx]

**Supplementary Table 1. Comparison of baseline characteristics between the current study and the pivotal trials: COPERNICUS and GALILEO**

| **Characteristic** | **Treat and Extend** | **COPERNICUS** | **GALILEO** |
| --- | --- | --- | --- |
| Age, years |  |  |  |
| Mean ± SD | 61.8 ± 14.3 | 65.5 ± 13.6 | 59.9 ± 12.4 |
| Gender, male (%) | 80% | 61% | 56.3% |
| BCVA |  |  |  |
| ETDRS letters | 55.0 [36.8 to 64.8] | 50.7 ± 13.9 | 53.6 ±15.8 |
| >20/200, (%) | 75% | 75.4% | 83.5% |
| CMT, μm |  |  |  |
| Mean ± SD | 807 ± 238 | 661.7 ± 237.4 | 683.2 ± 234.5 |
| IOP |  |  |  |
| Mean ± SD | 14.3 ± 2.6 | 15.1 ± 3.26 | 15.1 ± 2.8 |
| Perfusion Status |  |  |  |
| Perfused (%) | 50% | 67.5% | 86.4% |

SD: standard deviation; BCVA: best-corrected visual acuity; ETDRS: Early Treatment of Diabetic Retinopathy Study; CMT: central macular thickness; IOP: intraocular pressure
