# Supplementary material for: A treat and extend protocol with Aflibercept for cystoid macular oedema secondary to central retinal vein occlusion – an 18-month prospective cohort study
Source: BMC Ophthalmol. 2020 Feb 24;20:69. doi: 10.1186/s12886-020-01346-8 (PMC7038604; doi:10.1186/s12886-020-01346-8)
Supplement: Supplementary file 2 — Additional file 2. Supplementary Table 2. The Significance of Baseline Demographics and Study Eye Characteristics on Mean Change in Visual Acuity after 18 Months of Aflibercept Treatment. [file 12886_2020_1346_MOESM2_ESM.docx]

**Supplementary Table 2. The Significance of Baseline Demographics and Study Eye Characteristics on Mean Change in Visual Acuity after 18 Months of Aflibercept Treatment**

| **Characteristic** | **Odds ratio (95% Confidence Interval)** | **P Value** |
| --- | --- | --- |
| Age, years | -0.15 (-0.68 to 0.35) | 0.48 |
| Gender  Female vs. male | 0.02 (-20.16 to 21.93) | 0.92 |
| Hypertension | 0.13 (-13.95 to 22.08) | 0.61 |
| Diabetes | 0.14 (-16.98 to 26.18) | 0.63 |
| Hypercholesterolaemia | -0.33 (-30.84 to 4.53) | 0.12 |
| Baseline visual acuity | -0.90 (-1.37 to -0.45) | **<0.01** |
| Baseline central macular thickness^*^ | 0.00 (-0.32 to 0.32) | 0.99 |
| Baseline intraocular pressure | 0.19 (-1.70 to 4.17) | 0.35 |
| Baseline perfusion status | -0.09 (-11.84 to 8.34) | 0.69 |

^*^ Baseline central macular thickness was divided by 10. The resulting odds ratios was per 10 μm difference in baseline central macular thickness.
