# Supplementary figures and images for: A treat and extend protocol with Aflibercept for cystoid macular oedema secondary to central retinal vein occlusion – an 18-month prospective cohort study
Source: BMC Ophthalmol. 2020 Feb 24;20:69. doi: 10.1186/s12886-020-01346-8 (PMC7038604; doi:10.1186/s12886-020-01346-8)

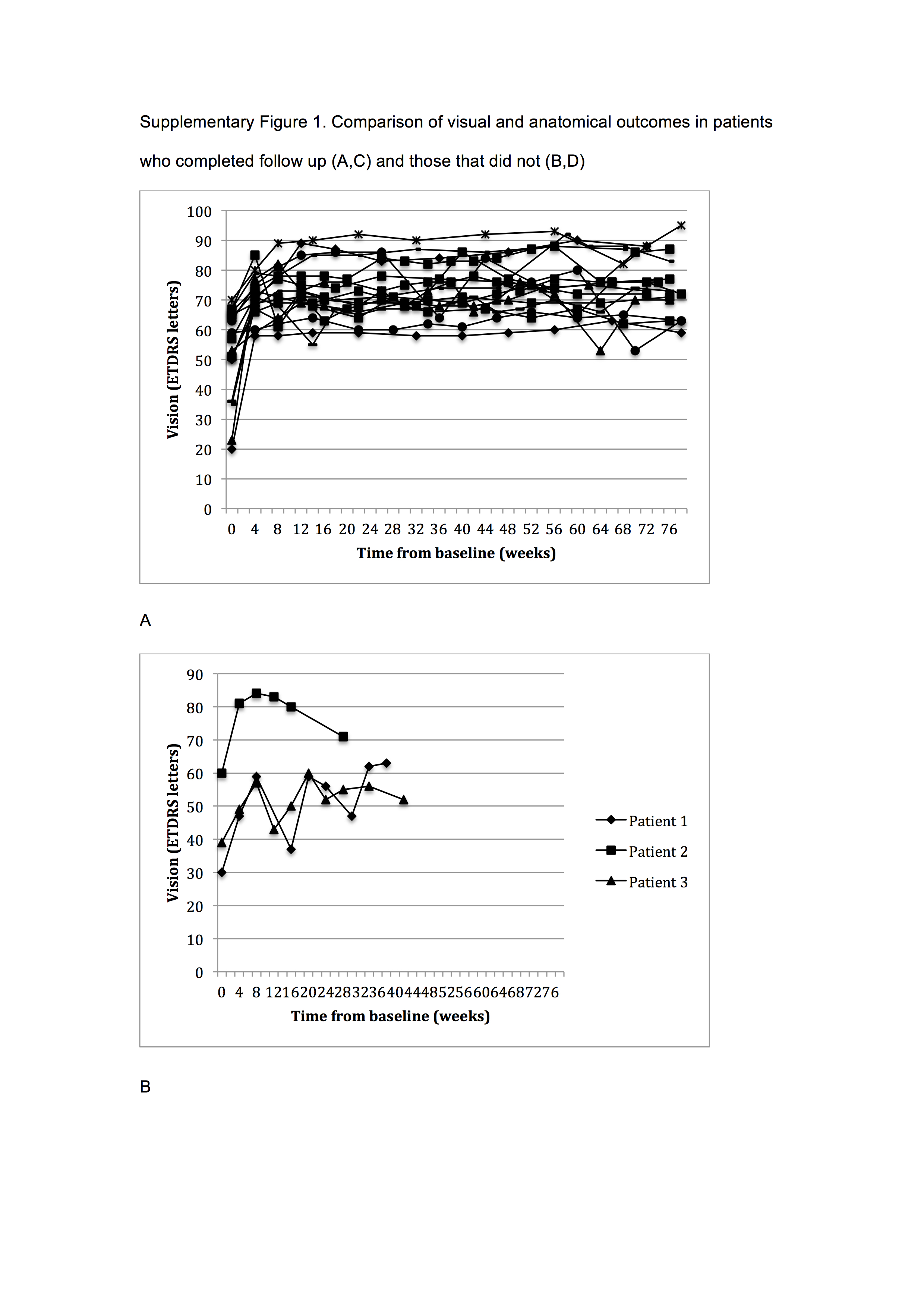

Supplement: Supplementary file 3 — Additional file 3. Supplementary Figure 1. Comparison of visual and anatomical outcomes in patients who completed follow up (A,C) and those that did not (B,D). [file 12886_2020_1346_MOESM3_ESM.jpg]
